# Delayed Diagnosis of Spinal Tuberculosis in a 44-year-old Male with Acute on Chronic Low Back Pain

**DOI:** 10.5811/cpcem.2018.11.38575

**Published:** 2019-04-02

**Authors:** Curt Canine, Sarah Medeck, Anthony Hackett

**Affiliations:** Carl R. Darnall Army Medical Center, Department of Emergency Medicine, Fort Hood, Texas

## Abstract

Spinal tuberculosis (STB), also known as tuberculous spondylitis, tuberculous vertebral osteomyelitis, or Pott’s disease is a rare subset of extrapulmonary tuberculosis. Although rare in developed countries, STB is an important diagnosis for the emergency physician to consider. We report a case of a 44-year-old African-American male with STB presenting as an acute exacerbation of chronic low back pain complicated by urinary retention and difficulty ambulating. Our patient had no known predisposing risk factors for tuberculosis. This patient’s STB was mistakenly diagnosed as nontuberculous vertebral osteomyelitis. This is not uncommon, as it is often difficult to distinguish the two clinically. This patient experienced advanced neurologic features at the time of initial presentation, which improved with surgical decompression. Ultimately, he re-presented to the emergency department 10 days after hospital discharge with recurrence of symptoms due to inaccurate antimicrobial selection. The diagnosis may hinge on the astute physician recognizing the characteristic, albeit subtle, imaging findings of STB.

## INTRODUCTION

Spinal tuberculosis (STB), also known as tuberculous spondylitis, tuberculous vertebral osteomyelitis, or Pott’s disease, is a rare subset of extrapulmonary tuberculosis (TB), which is itself a rare disease in immunocompetent patients. In the 10-year period from 2002 and 2011 there was an average of one case of STB per two million persons in the United States (U.S.).^14^ Despite ongoing efforts by the World Health Organization and the United Nations to eradicate TB, 10.4 million new cases were diagnosed worldwide in 2015. The U.S. alone saw 10,000 new TB cases in 2015, a 20% increase from 2014. TB remains among the top 10 causes of death worldwide with a global case fatality rate of 17% in 2015.^11^

## CASE REPORT

**Image 1:**
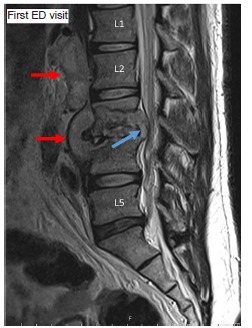
Pre-operative sagittal magnetic resonance imaging from index visit demonstrates bony destruction of third and fourth lumbar vertebrae. A large epidural abscess extending from first to fourth lumbar vertebrae (red arrows) projects through third lumbar vertebral body and compresses the spinal nerve roots (blue arrow). Lumbar vertebrae 1 (L1), 2 (L2), and 5 (L5). *ED*, emergency department.

A 44-year-old African-American male with a history of chronic low back pain presented to the emergency department (ED) having difficulty walking and trouble urinating. He reported no classic TB risk factors and denied history of international travel or exposure to high-risk populations. He also denied a history of intravenous (IV) drug use. Nor did he report typical preceding symptoms of night sweats, fever, weight loss, cough, or hemoptysis. Initial vital signs included blood pressure 123/85 millimeters of mercury (mmHg), pulse 127 beats per minute (bpm), respirations 28 per minute, oxygen saturation 99% on room air, and afebrile at 98.1° Fahrenheit (F). Due to concern for possible cauda equina syndrome, emergent magnetic resonance imaging (MRI) of the lumbar spine was done (Image 1). The patient was diagnosed with discitis, osteomyelitis and ventral epidural abscess at lumbar vertebrae 3 and 4 (L3, L4). Labs revealed leukocyte count 8.6 x10^9^ per liter (L), hemoglobin 12.4 grams per deciliter (g/dL), platelets 319 x10^9^/L, C-reactive protein (CRP) 1.15 milligrams (mg)/dL, erythrocyte sedimentation rate 56 millimeters per hour and lactic acid 0.8 millimoles/L. Urine drug screen, hepatitis panel, human immunodeficiency virus screen and rapid plasmin reagin test all returned negative.

He was promptly transferred to a hospital with neurosurgical capabilities and taken to the operating room for L3 laminectomy with partial facetectomy and evacuation of the ventral epidural abscess. Successful decompression of the L3 and L4 nerve roots was achieved, and abscess fluid was sent for culture. The patient was admitted and started on broad-spectrum IV antibiotics. Culture results from the epidural abscess returned *Propionibacterium acnes*, while the pathology report was negative for fungal elements. The acid-fast bacilli (AFB) stain was also negative. Antibiotic coverage was narrowed to ceftriaxone only. After eight days in the hospital, the patient improved significantly, ambulating without difficulty, tolerating physical and occupational therapy. A peripherally inserted central line was placed before discharge to home, and arrangements were made for weekly lab monitoring and home IV ceftriaxone therapy. A prednisone taper was also prescribed for lingering radicular pain.

**Image 2:**
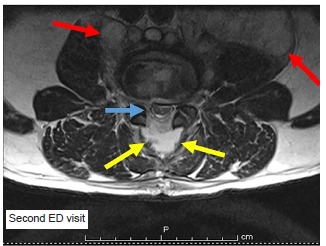
Axial magnetic resonance image at the level of the third lumbar vertebrae taken on the second visit demonstrates progression of ventral epidural abscess now extending to completely surround the lumbar nerve roots (blue arrow), and extending into posterior vertebral space (yellow arrows). Again the left psoas muscle demonstrates inflammatory changes concerning for abscess with interval progression of right psoas muscle inflammation (red arrows). *ED*, emergency department.

**Image 3:**
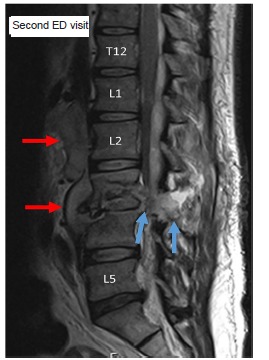
Midline sagittal magnetic resonance image taken on second visit re-demonstrates epidural abscess (red arrows) that has spread to extensively involve posterior vertebral soft tissue elements (blue arrows). Lumbar vertebrae 1 (L1), 2 (L2), and 5 (L5). Thoracic vertebrae 12 (T12). *ED*, emergency department.

Ten days after discharge the patient presented once again to the ED for progressively worsening lumbar and thoracic back pain, lower extremity weakness and inability to pass a bowel movement. Initial vital signs were blood pressure 127/90 mmHg, pulse 102 bpm, respirations 21 per minute, oxygen saturation 98% on room air, and afebrile at 97.9° F. A second MRI was emergently ordered (Images 2 and 3), which demonstrated interval progression of infectious changes involving L3 and L4 with associated epidural and retroperitoneal spread of infection. Given the report of upper back pain on this second ED visit the MRI also imaged the thoracic spine, which identified extensive, abnormal epidural enhancement throughout the thoracic spinal canal suggestive of diffuse infection, with focal epidural thoracic spine abscess formation identified at vertebral levels 4–7.

Chart review revealed a positive QuantiFERON®-TB Gold serum test, as well as a culture positive for *Mycobacterium tuberculosis* from the initial lumbar epidural abscess drained during decompressive neurosurgery. Vital signs were unremarkable with blood pressure 119/72 mmHg, pulse 94 bpm, respirations 18 per minute, oxygen saturation 98% on room air, and afebrile at 97.9° F. Physical examination was significant only for 4/5 strength to bilateral lower extremities. Significant lab abnormalities included critically low potassium 2.7 milliequivalents/L, leukocyte count 10.8 x10^9^/L, platelets 370 x10^9^/L and CRP 10.36 mg/dL. The patient was admitted to a negative-pressure isolation bed and started on rifampin, isoniazid, pyridoxine, pyrazinamide and ethambutol for TB. The original neurosurgery team evaluated the patient again on the second hospital admission and determined no further operative intervention was necessary. The Department of Health was notified of the positive TB result and the patient was discharged home on hospital day eight on a monitored four-drug antibiotic regimen with a diagnosis of retroperitoneal abscess due to extension of Pott’s disease of the spine.

**CPC-EM Capsule**

What do we already know about this clinical entity?

*Spinal tuberculosis (STB) is a rare disease with a prevalence of one in two million in the west. With the rise in TB cases worldwide, STB may become more common*.

What makes this presentation of disease reportable?

*This case underscores the importance of expanding one’s differential diagnosis for spinal epidural abscess (SEA) even in the immunocompetent host*.

What is the major learning point?

*STB can be present in an an immunocompetent host. It should be considered especially if a patient returns with similar symptoms after effective treatment of SEA*.

How might this improve emergency medicine practice?

*Readers should be able to recognize the pearls and pitfalls of making this diagnosis and the possibility that STB may exist in an immunocompetent host*.

## DISCUSSION

The most common presenting symptoms in STB are back pain (70.4%), weight loss (30.3%), neurologic abnormalities (30.1%), and night sweats (19.1%).^3^ A characteristic “aldermanic gait” describes patients taking short, deliberate steps to avoid jarring the spine. Among those presenting with neurologic symptoms, limb weakness was most common (33%), followed by complete limb paralysis (15%), and urinary or fecal incontinence (8%).^1^ Time from initial symptoms to diagnosis is often quite protracted, with reports ranging from 78 days to over 1.5 years.^2^,^3^ Half of STB patients present with isolated back pain and no other symptoms at the time of diagnosis, contributing to an initial misdiagnosis rate of 41%.^4^,^5^ Further confounding the diagnosis, clinical judgment is shown to be insensitive and poorly specific.^6^

A panel of clinicians, laboratory scientists, and TB control experts from the Centers for Disease Control and Prevention and the Association for Public Health Laboratories, convened in 2008 and proposed a diagnostic algorithm for the diagnosis of TB, which pairs nucleic acid amplification testing (NAAT) results (positive or negative) with AFB smear results (positive or negative) to dictate treatment. A positive NAAT and positive AFB smear is presumed TB positive, antibiotics started and cultures followed. Unfortunately, all other combinations of results are indeterminate, and the recommendation is to use clinical judgment, repeat NAAT and AFB smear testing, and wait for culture results. While NAAT results are available within 48 hours and have a positive predictive value of 95%, this algorithm relies on AFB smear, which has been shown to be poorly sensitive, may require repeat sampling, and is dependent on the quality of the sample obtained.

Further challenging its utility in the diagnosis of TB, NAAT performs poorly as a screening test in patients who have low clinical probability of TB.^7^ QuantiFERON-TB Gold serum test is an interferon-gamma release assay (IGRA) that measures T-cell release of interferon-gamma following stimulation by antigens unique to *M. tuberculosis* and a few other mycobacteria. IGRAs cannot distinguish between latent infection and active TB disease and should not be used for diagnosis of active TB, as a negative IGRA does not rule out active TB.^8^ Several newer, rapid, biomarker assays are currently under development, promising sensitivities nearing 100% with short turn-around times compared to traditional culture-based methods, but these assays require further evaluation before they are ready for clinical use.^3^

STB typically develops from hematogenous spread from lung to lumbar or lower thoracic vertebrae, via the venous plexus and then spreading to adjacent vertebrae under the anterior longitudinal ligament and destroying anterior vertebral elements while preserving intervertebral discs and posterior elements. When many vertebrae are affected, a characteristic, sharply-angulated thoracolumbar kyphosis develops. This finding is known as a gibbus deformity (not seen in this case). These features distinguish STB from nontuberculous vertebral osteomyelitis, which affects the intervertebral discs early and vertebral elements more equally. In the case of STB, as opposed to nontuberculous osteomyelitis, paraspinal abscess is quite common at time of presentation.^9^,^3^ At the time of STB diagnosis the majority of patients were found to have MRI evidence of inflammatory paraspinal tissue changes (89.7%) or thecal compression (93.1%).^10^

Significant bone mineral loss (30%) is required before radiolucent lesions appear on plain films, a finding often delayed up to eight weeks.^3^–^4^ Chest radiograph (CXR) should be obtained when a diagnosis of STB is entertained, but it provides little help in diagnosing STB as approximately half will show no lesion. CXR revealed evidence of active or healed pulmonary disease in 30% of STB patients at time of diagnosis. Plain films of the spine may show endplate disruption and bone destruction but little else.^4^

Computed tomography is excellent at demonstrating bony lytic lesions, sclerosis, bone circumference disruption, disc space collapse, and paraspinal abscess with rim enhancement.^4^ However, MRI is the imaging modality of choice as it superiorly demonstrates soft tissue involvement, epidural extension, and involvement of neural structures, often before onset of clinical symptoms.^3^,^2^,^11^

Untreated TB mortality is 70% at 10 years, and early diagnosis is paramount. STB should be treated for two months with quadruple therapy, usually rifampin, isoniazid, pyrazinamide and ethambutol, then rifampin and isoniazid continued for a total of six months. Children should be treated for one year.^12^ For persistent symptoms, and lab or imaging signs of continued infection, options include extending medical therapy through nine months or operative management.^11^ Surgical intervention is indicated in patients with compression of the spinal cord or nerve roots, spinal deformity leading to instability, extensive abscess formation, or medical treatment failure. One large study showed 37% of STB patients required operative management, and 10% of those required multiple surgeries.^11^,^2^

## CONCLUSION

This patient’s STB was mistakenly diagnosed as nontuberculous vertebral osteomyelitis. This is not uncommon as it is often difficult to distinguish the two clinically. Both present with lumbar pain, have similar imaging findings and no proven diagnostic laboratory criteria with rapid results. It is not surprising then that STB diagnosis is often delayed months to years, increasing the chance of developing kyphotic deformities, neurologic deficits, and increased need for surgical management at the time of diagnosis.^4^,^5^,^13^ This patient experienced advanced neurologic features at the time of initial presentation, which improved with surgical decompression but ultimately returned due to inaccurate antimicrobial selection.

The diagnosis may hinge on the astute physician recognizing the characteristic, albeit subtle, imaging findings of STB. These include: 1) anterior vertebral body element destruction with preservation of intervertebral discs and posterior vertebral elements of contiguous lumbar or lower thoracic vertebrae; 2) concomitant psoas-muscle abscess formation (often quite extensive); and later 3) gibbus and kyphotic deformities. Additionally, clues based on the clinical history as well as the presence of refractory spinal epidural symptoms despite culture-guided therapy should lend clues to the diagnosis.
